# Terror Medicine as Part of the Medical School Curriculum

**DOI:** 10.3389/fpubh.2014.00138

**Published:** 2014-09-12

**Authors:** Leonard A. Cole, Katherine Wagner, Sandra Scott, Nancy D. Connell, Arthur Cooper, Cheryl Ann Kennedy, Brenda Natal, Sangeeta Lamba

**Affiliations:** ^1^Rutgers New Jersey Medical School, Newark, NJ, USA; ^2^Hofstra North Shore-LIJ School of Medicine, Hempstead, NY, USA; ^3^Columbia University Medical Center at Harlem Hospital, New York, NY, USA

**Keywords:** terror medicine, medical education, preparedness, disaster medicine, emergency planning

## Abstract

Terror medicine, a field related to emergency and disaster medicine, focuses on medical issues ranging from preparedness to psychological manifestations specifically associated with terrorist attacks. Calls to teach aspects of the subject in American medical schools surged after the 2001 jetliner and anthrax attacks. Although the threat of terrorism persists, terror medicine is still addressed erratically if at all in most medical schools. This paper suggests a template for incorporating the subject throughout a 4-year medical curriculum. The instructional framework culminates in a short course for fourth year students, such as one recently introduced at Rutgers New Jersey Medical School, Newark, NJ, USA. The proposed 4-year Rutgers curriculum serves as a model that could assist other medical schools contemplating the inclusion of terror medicine in pre-clerkship and clerkship training.

The 1995 Oklahoma City bombing, the September 11, 2001 attacks, the subsequent anthrax letters, and the 2013 Boston Marathon bombings demonstrate that the United States homeland continues to be a target of terrorists. Physicians and other medical personnel have particular responsibilities in such events. Their roles are part of terror medicine, which encompasses four broad areas: preparedness, incident management, mechanisms of injuries, and psychological consequences (1). While terror medicine incorporates aspects of disaster and emergency medicine, it also has distinctive features. It includes, for example, recognizing “the signs and symptoms specific to particular kinds of terrorist weapons,” such as the biological agent *Bacillus anthracis* (the cause of anthrax) and the chemical agent sarin, and using “management principles” that are geared toward managing an entire event, not just a single patient (2).

## The Gap between Need and Implementation

Following 9/11, medical schools and teaching hospitals reacted “with a flurry of special programs on emergency medicine and anthrax exposure” (3). Some medical leaders urged the incorporation of “terrorism preparedness and response material into the curricula for every health professions school in the nation” (4). Deborah Danoff, associate vice president of the Association of American Medical Colleges (AAMC), called for “a long-term plan” that would address this newly acknowledged need (3).

More than a decade later, however, the widespread sense of urgency for curricular change has dissipated. Even the longer-established and related field of disaster medicine is taught at less than one-third of United States medical schools (5). What every medical school is doing to prepare its students is difficult to determine, because each has considerable flexibility in designing its own pre-clerkship and clerkship curricula. However, the larger picture may be assessed by a review of the requirements that American and Canadian schools must meet. Further, the curricula of select individual schools can provide insight at the local level. The resulting impression is that courses explicitly on terror medicine, such as the one recently introduced at Rutgers New Jersey Medical School (NJMS) (6) are offered at few institutions. The Rutgers elective (for fourth year students) comprises exercises and presentations on key areas of terror medicine including the use of biological and chemical agents, incident management, traumatic injury, and psychological effects.

Accreditation for American and Canadian schools is required every few years by the Liaison Committee on Medical Education (LCME). An institution must pass a site review and respond to any critiques with acceptable revisions, though it is not otherwise required to alter its training plans for future physicians. In 2013, the LCME released a report on the standards required for a school to maintain its accreditation in the next 2 years (7). Nine areas are listed under “Standard 7: Curricular Content”:

1. Biomedical, behavioral, social sciences
2. Organ systems/life cycle/primary care/prevention/wellness/symptoms/signs/differential diagnosis, treatment planning, impact of behavioral/social factors
3. Scientific method/clinical/translational research
4. Critical judgment/problem-solving skills
5. Societal problems
6. Cultural competence/health care disparities/personal bias
7. Medical ethics
8. Communication skills
9. Interprofessional collaborative skills

Not only is reference to terror medicine absent from the document, so are the words “terrorism,” “radiation,” “nuclear,” “bioweapon,” and similarly relevant terms. Even in 2002, when distress about 9/11 was still fresh, the LCME had no “active” plans to improve terror medicine education (3). The inclusion of terror medicine in LCME requirements would provide an incentive for medical schools, much as the recently added interprofessional education requirement has spurred a growth in that domain. Without LCME specification, however, there is little impetus for medical schools to ensure that students learn anything about terror medicine basics. Including a course on the subject requires individual schools to go above and beyond the LCME accreditation standards.

While there is no national push to incorporate terror medicine into medical education, individual schools have the ability to supplement the required curriculum with mandatory and elective coursework. New York City area schools would seem among the most likely candidates for such efforts in light of the September 11, 2001 attacks and the subsequent anthrax-laden letters mailed in that region. Robert Holzman, who was running New York University’s Langone School of Medicine’s (LSOM) Center for Health Information and Preparedness, reported soon after that his institution might incorporate information about chemical and biological weapons and the response to terrorist events into its mandatory course on “The Physician, Patient, and Society” (3). In 2010, the LSOM introduced the Curriculum for the twenty-first Century (C21).

The school’s current website explains that C21 includes “ongoing educational exercises called pillars that span the 4 years of medical school.” Topics that students might pursue for 4 years include important primary health care issues like diabetes, atherosclerosis, and colon cancer, and infectious disease issues like tuberculosis. While the curriculum may include bits and pieces of information about terrorism, there is no overt emphasis on it (8).

Another means of assessing interest in terror medicine at a particular institution is by looking at the Grand Rounds schedule (links to schedules are listed before the reference section). Although Grand Rounds and department conferences are not the largest portion of the curriculum, the topic/speaker selection is indicative of what faculty think is salient issues about which students, residents, and physicians should be educated. The LSOM Department of Medicine’s 67 Grand Rounds between September 2011 and June 2013 included only one topic and speaker devoted to any aspects of terrorism. On October 11, 2011, a decade after the 2001 attacks, Anna Nolan presented information about World Trade Center Lung Injury.

The Emergency Medicine Grand Rounds schedule for New York Presbyterian Hospital, which is affiliated with both Columbia University and Cornell University, shows one presentation on blast injuries and one on toxicology since 2006. The department instead has emphasized the legal aspects of emergency medicine, as three different speakers have spoken about lawsuits. The Department of Medicine at the Icahn School of Medicine/Mount Sinai in New York listed no presentations about terror-related subjects (its archives show talks since 2011). The Department of Emergency Medicine at Mount Sinai’s website cites one talk about disaster surge capacity, which was given in 2009, and little other mention of terror or disaster medicine.

Prior to the new 2014 elective course on terror medicine at the Rutgers NJMS, attention to the subject was sparse. Even in light of Hurricane Irene and Hurricane Sandy, which devastated parts of New York and New Jersey, there has been little discussion about disaster preparedness, a topic that dovetails with terror medicine and general preparedness at the aforementioned institutions.

In fact, aspects of terror medicine apply as well to natural or accidentally induced incidents. Treatment of the victim of a fire or a chemical or biological exposure adheres to certain principles whether or not the cause was deliberate. Thus, while the focus of discussion may be terror-related, its academic value extends beyond.

Of course, terror- and disaster-related subjects might have been discussed in forums other than those reviewed here. But the low priority that accorded these matters is evident by their absence from key educational venues at these institutions.

## Opportunities during Medical Education

Fortunately, there are simple ways to ensure that medical students and residents have exposure to terror medicine and are, at the very least, introduced to its principles. The medical curriculum offers ample opportunity to discuss the response efforts in terror and other disaster incidents. The required basic science courses and clinical clerkships can integrate some of the basics quite easily. Following are possible portals for incorporating this material into the 2015 Rutgers NJMS curriculum, now being considered, and which may provide a template for other institutions as well.

When planning for incorporation of new content or teaching new skills, three levels can be envisioned: exposure, immersion, and competence. Most medical school curricula have a similar three-phase set-up, as is also planned for the 2015 NJMS curriculum. The first phase starts with orientation followed by foundational courses and organ systems teaching; the second phase includes core third year clerkships; the third phase provides fourth year student concentrations on key areas of future growth. The terror medicine curriculum would follow these phases: first, exposure to the discipline; second, immersion in specific areas of interest; and third, opportunities to gain competence and deepen understanding of the field.

## Phase 1: Exposure

### Orientation

New Jersey Medical School, like many other medical schools, devotes at least 1 week to orientation for first year students. This presents an initial opportunity to describe first responder tasks associated with emergencies, terrorist events, and other disasters. A discussion of the National Incident Management System could be included (9). NJMS is fortunate to have numerous first responders based at University Hospital who can familiarize medical students with their field. Emergency Medical Technicians (EMTs) already teach clinical emergency assessment during Phase 1, which introduces students to different forms of personal protection equipment (PPE) besides the gowns and gloves that are ubiquitous in hospital settings. In fact at Hofstra Medical School in Hempstead, NY, USA, the first year curriculum already includes EMT training for which every medical student receives certification (10).

### Foundations block

Medical school curricula commonly begin with a foundation in biochemistry, genetics, and microbiology. This block is opportune for considering the disruptive effects of sarin and other nerve agents on cell receptors, as well as discussion of microbial weapons like anthrax. Lecturers can discuss briefly the anthrax letters and subsequent investigation and findings, along with the 1995 release of sarin in the Tokyo subway and more recently in Syria. Mentioning these agents at this point foretells later classes in pharmacology when treatments can be covered more comprehensively.

### Organ systems block

This block includes the teaching of anatomy, physiology and pathophysiology, and pharmacology. Radiation therapy is discussed in the unit on cancer, and radiation as a weapon could also be referenced during this unit. Information on response to a radiological attack would include methods of protection and treatments/therapies for survivors. Faculty could also advise students on the availability of additional courses online, for example, FEMA’s tutorial on radiological/nuclear training (11). Cholinergic and anticholinergic agents are usually discussed in pharmacology content, where atropine and 2-PAM (2-pyridine aldoxime methyl chloride) can be reviewed as countermeasures to exposure to organophosphates such as the nerve agents sarin, tabun, and VX. Similarly, lectures on antimicrobial therapies can reinforce the bioweapons discussions provided in the Foundations block.

## Phase 2: Immersion

### Third year clerkships

The Surgery Department at Rutgers NJMS has traditionally and effectively taught about blast injuries, burn, and other physical traumas. Mass casualty discussions would be a natural fit to this content. The psychiatry clerkship provides an opportunity to consider post-traumatic stress and the more intensified psychological effects caused by terror attacks compared to accidental or naturally occurring events. The surgery and psychiatry departments at NJMS as at many other American medical schools benefit from faculty members who previously have treated battlefield casualties or victims of terrorism and other disasters.

## Phase 3: Opportunities for Concentration and Competence

### Fourth year clerkships

The fourth year Emergency Medicine clerkship, for example, reviews some toxicology and could offer content as well as hands-on training with manikins programed as victims of chemical, biological, or radiation exposures. Students are thus able to participate in drills and treat victims of simulated terrorist attacks with various weapons of mass destruction.

Another example at NJMS is the Preventive Medicine clerkship, which includes the teaching of population health, biostatistics, and epidemiology. As at other schools that offer these subjects, they are also portals for terror medicine issues. Here, the role of the Centers for Disease Control and Prevention may be underscored as the key national agency that establishes protocols on preparedness for and response to a biological or chemical weapon exposure. A course such as the terror medicine elective at NJMS then provides opportunities for further knowledge and experience in the field.

Along with providing content, assessment of knowledge should occur at each phase of the curriculum. Throughout all stages of medical training, the Simulation Center can be particularly useful to practice in a safe learning environment. Simulated settings provide opportunities for ongoing feedback from learners as well as enabling assessment of competence.

How often an individual must participate in exercises for lasting benefit remains unclear. One study indicated that a successful drill improved participants’ knowledge base, but that the enhanced effect had diminished a year later (12). Another report found that 1 year after the 2013 Boston bombings, experience at that event still remained a valuable platform for effective preparedness (13).

In any case, presenting aspects of terror medicine throughout the 4 years of medical school – building knowledge levels and ending with a concentrated short course – increases the likelihood that a student’s retention and response capabilities will be long-term.

Students and teachers who participated in the new 2-week elective at NJMS, which ranged from key elements of preparedness to the psychological effects of terrorism, were uniformly enthusiastic about the experience (14). Student evaluations variously described the course as “excellent” and “amazing.” One student noted that she previously had felt unprepared to be helpful in the event of a terror or disaster incident. Even though embarking on a residency in a non-acute response specialty, she recognized that any physician could face such a circumstance. After finishing the course she indicated that she could no longer say, “Oh, I don’t handle this – sorry.”

## Conclusion

Decade-old proposals that aspects of terror medicine be incorporated into the curricula at American medical schools have been heeded erratically. Yet, the continuing threat of terrorism calls for the preparation of future physicians in the fundamentals of this field. As demonstrated at Rutgers in 2014, student and faculty interest in the subject is palpable. The instructional format presented here could assist medical schools that plan to incorporate terror medicine into their curricula.

## Grand Rounds Schedules

Department of Medicine. *New York University Grand Rounds*. (2014). Available from: http://medicine.med.nyu.edu/education/grand-rounds

Department of Medicine. *Rutgers New Jersey Medical School*. New Jersey (2014). Available from: http://njms.rutgers.edu/departments/medicine/grandrounds.cfm

Grand Rounds Speakers. *New York Presbyterian Emergency Medicine*. (2014). Available from: http://nypemergency.org/residency/grand_round_speakers.html?name1=Grand+Round+Speakers&type1=2Active#

Institute for Medical Education. *Icahn School of Medicine at Mount Sinai*. New York Available from: http://icahn.mssm.edu/education/institute-for-medical-education/programs-courses-and-events/grand-rounds

Lectures. *Sinai EM*. New York (2014). Available from: http://www.sinaiem.org/academics/lectures/

## Conflict of Interest Statement

The authors declare that the research was conducted in the absence of any commercial or financial relationships that could be construed as a potential conflict of interest.
